# Characterisation of long-term cancer survivors and application of statistical cure models: a protocol for an observational follow-up study in patients with colorectal cancer

**DOI:** 10.1186/s12889-020-09807-x

**Published:** 2020-11-17

**Authors:** Sonia Pértega-Díaz, Vanesa Balboa-Barreiro, Rocío Seijo-Bestilleiro, Cristina González-Martín, Remedios Pardeiro-Pértega, Loreto Yáñez-González-Dopeso, Teresa García-Rodríguez, Teresa Seoane-Pillado

**Affiliations:** 1 Research Support Unit, Nursing and Healthcare Research Group, Rheumatology and Health Research Group, Instituto de Investigación Biomédica de A Coruña (INIBIC), Complexo Hospitalario Universitario de A Coruña (CHUAC), Sergas, Universidade da Coruña (UDC), As Xubias, Hotel de Pacientes 7ª Planta, 15006 A Coruña, Spain; 2 Digestive Apparatus Service, Complexo Hospitalario Universitario de A Coruña (CHUAC), Sergas, As Xubias, 15006 A Coruña, Spain

**Keywords:** Colorectal neoplasms, Long-term survivors, Quality of life, Prognostic factors, Cure

## Abstract

**Background:**

Improved colorectal cancer (CRC) survival rates have been reported over the last years, with more than half of these patients surviving more than 5 years after the initial diagnosis. Better understanding these so-called long-term survivors could be very useful to further improve their prognosis as well as to detect other problems that may cause a significant deterioration in their health-related quality of life (HRQoL). Cure models provide novel statistical tools to better estimate the long-term survival rate for cancer and to identify characteristics that are differentially associated with a short or long-term prognosis. The aim of this study will be to investigate the long-term prognosis of CRC patients, characterise long-term CRC survivors and their HRQoL, and demonstrate the utility of statistical cure models to analyse survival and other associated factors in these patients.

**Methods:**

This is a single-centre, ambispective, observational follow-up study in a cohort of *n* = 1945 patients with CRC diagnosed between 2006 and 2013. A HRQoL sub-study will be performed in the survivors of a subset of *n* = 485 CRC patients for which baseline HRQoL data from the time of their diagnosis is already available. Information obtained from interviews and the clinical records for each patient in the cohort is already available in a computerised database from previous studies. This data includes sociodemographic characteristics, family history of cancer, comorbidities, perceived symptoms, tumour characteristics at diagnosis, type of treatment, and diagnosis and treatment delay intervals. For the follow-up, information regarding local recurrences, development of metastases, new tumours, and mortality will be updated using hospital records. The HRQoL for long-term survivors will be assessed with the EORTC QLQ-C30 and QLQ-CR29 questionnaires.

An analysis of global and specific survival (competitive risk models) will be performed. Relative survival will be estimated and mixture cure models will be applied. Finally, HRQoL will be analysed through multivariate regression models.

**Discussion:**

We expect the results from this study to help us to more accurately determine the long-term survival of CRC, identify the needs and clinical situation of long-term CRC survivors, and could be used to propose new models of care for the follow-up of CRC patients.

## Background

Cancer is one of the most frequent diseases worldwide and is one of the main causes of hospital admissions. According to GLOBOCAN data, there were a total of 18.1 million new cancer cases and 9.6 million cancer deaths in 2018 [1]. In both sexes combined, colorectal cancer (CRC) is the fourth most commonly diagnosed cancer and the fifth leading cause of cancer-related death [1]. Advances in the diagnosis and treatment of cancer patients have generally increased patient survival rates. Indeed, according to the American Cancer Society (ACS), the 5-year survival rate after a cancer diagnosis is around 65% for all tumours, as well as for CRC in particular [2]. EUROCARE-5 data indicates that in Europe, the survival rate 5 years after diagnosis exceeds 50% for most tumour sites [3]. According to this report, the age-standardised 5-year survival rate for colon cancer was 57 and 55.8% for rectal tumours [3].

Despite these figures, very few prospective studies have evaluated the recurrence or mortality rates in survivors of CRC [4]. Although controversial, a patient with an oncological process diagnosis is currently considered ‘cured’ when they survive 5 years after diagnosis [5], with these patients often being referred to as ‘long-term survivors’. Thus, in CRC, standard follow-up strategies are usually performed periodically during this period [6]. However, other authors suggest that this ‘cure’ is not guaranteed for patients who survive the first 5 years after diagnosis and that follow-up strategies should be modified according to the risk factors presented by each patient [4]. Thus, it would be useful to use a rigorous methodology to determine, in greater detail, the probability (or percentage) of cured patients, i.e. long-term CRC survivors, as well as the variables associated with the prognosis of both these and ‘non-cured’ patients in order to establish more appropriate follow-up strategies.

Kaplan–Meier curves and the Cox proportional hazard models are the statistical methods most commonly used to analyse all-cause mortality in cancer studies, while a competing-risk analysis is preferred to determine cause-specific mortality and its associated factors [7]. However, cure models, which are still not often used, provide an alternative statistical tool to estimate the cure rates of cancer patients and analyse the differences between those individuals who are long and short-term survivors, as well as to identify covariates associated differently with short or long-term progosis [8]. Although some authors have highlighted the usefulness of cure models as an analysis strategy which could provide especially useful information for quantifying the improvement in survival figures in CRC [9], very few publications are available in this regard. These studies show cure rates of around 50% and a median survival rate for uncured patients of about 1 year after diagnosis [9–13]. No such studies have been undertaken in Spain, and furthermore, most of this work has been carried out using population registries and/or do not include clinical information other than the age at diagnosis, sex, and disease stage, and therefore, have not used these models to explore the impact of other covariates on cure rates or survival time.

Data evaluating the long-term health-related quality of life (HRQoL) of CRC survivors are also scarce, although work studying the first 5 years after their diagnosis is more common [14]. Recent work suggests that these patients present a HRQoL similar to that of the general population, although some factors such as intestinal dysfunction can contribute to their deterioration, even 15 years after their initial diagnosis [14]. Furthermore, these results should be contrasted with those obtained in other cohorts and in different locations. Therefore, this study was planned with the aim of determining the long-term prognosis in CRC patients, to characterise long-term survivors and their HRQoL, and demonstrate the utility of statistical cure models in the study of CRC survival. Results from this study may be useful to determine the cure rate (proportion of long-term survivors) in CRC, the survival rates of ‘uncured’ patients and associated factors, and to develop a predictive model to identify long-term survivors from among CRC patients. In addition, it will also allow us to describe HRQoL and the prevalence of symptoms in these patients.

## Methods/design

### Objectives

The goals of this project are

i. To determine the long-term survival rates of CRC patients and the variables associated with the prognosis.
ii. To ascertain the cure rate (proportion of long-term survivors) for CRC and the survival rate for uncured patients as well as the factors associated with these by using mixture cure models.
iii. To develop a predictive model to identify long-term survivors from among patients diagnosed with CRC.
iv. To determine the HRQoL and prevalence of symptoms in long-term survivors of CRC.

### Design

This is a single-centre follow-up observational study of cases of CRC diagnosed in the Complexo Hospitalario Univesitario A Coruña (A Coruña, northwest Spain) from 2006 to 2013 (*n* = 1945), previously included in some of our other research projects [15–17]. A quality of life sub-study will be carried out in the patients included in the framework of one of these other projects [17] who have not died at the time of this present study (*n* = 485), in order to assess their current HRQoL and compare it with their baseline HRQoL scores when the CRC was initially diagnosed.

### Inclusion and exclusion criteria

Every case of CRC diagnosed during the 2006–2013 period was included. We excluded prevalent or recurrent cases, multiple cancer cases, cases detected by CRC screening, and cases diagnosed in another hospital but referred to our centre for treatment.

### Sample size

A cohort of *n* = 1945 incident CRC cases is available from previous research projects. Assuming exposure of 50% and a censored data percentage of 58% [3, 18], this sample size will make it possible to use a Cox regression model to detect significant changes with a hazard ratio (HR) of 1.22 or more (security = 95%; statistical power = 80%). Based on the available survival figures of the *n* = 485 patients included in a previous project for whom baseline HRQoL data are available [17], we estimate that around *n* = 281 patients will still be alive at the time of this proposed study. Assuming loses of around 30%, to estimate HRQoL with 95% confidence and at ±7% precision, we estimate that some *n* = 200 patients will need to be included in this HRQoL sub-project.

### Data collection

Cases were identified via the Pathological Anatomy Service at our hospital and the specialists who followed-up the patients were also responsible for their recruitment. After signing their informed consent to participation, we interviewed the patients and reviewed their primary and specialised care clinical history. We already have a computerised database in which we have collected the information from the framework of our other research projects [15–17]. In the case of the HRQoL sub-study, non-deceased patients will be informed of the objectives of this present study during one of their follow-up visits to the hospital. The HRQoL questionnaires will be completed after they grant their informed consent to participation in this new study.

### Measurements

Information about each patient was obtained in our previous studies and is detailed in our other publications [15–17]. This data includes

- Sociodemographic data: age at the time of the CRC diagnosis, sex, civil status, and education level.
- Patient interview data: perceptions and attitudes to the first symptoms of CRC.
- Personal or family history of cancer or other neoplasms.
- Comorbidities (Charlson comorbidity index).
- Disease presentation variables: first symptoms/signs, access to primary and specialised care, first consultation service, examinations and complementary tests prior to the diagnosis, laboratory tests at the time of the diagnosis.
- Diagnosis and treatment delay.
- Tumour characteristics at the time of diagnosis: tumour size, site, histological grade, macroscopic appearance, permeation, stage (TNM and Dukes), location of metastases, and infiltration of adjacent organs.
- Surgical treatment: curative or palliative surgery, type of surgery, access route (laparoscopic vs. open CRC surgery), type of anastomosis, resection of other viscera, resectability, surgical border infiltration, surgical morbidity, and reinterventions and their causes.
- Chemotherapy or radiation treatments.
- Complementary tests during follow-up: hospital consultations and physical examinations, laboratory tests, chest X-rays, abdominal ultrasound, colonoscopies, computed tomography, barium enema, and positron emission tomography.

The follow-up data that will be updated in this project include mortality (global and specific), local recurrences, distant metastases, and new-onset neoplasms, together with the date of each of these events. Non-deceased patients will be censored at the end of follow-up or on the latest date for which evidence is available for that patient.

In addition to the data described above, we will also record information about the HRQoL for the patients in the quality of life sub-study (*n* = 485 CRC patients diagnosed from 2010 to 2013) 3 to 6 months from the time of the CRC diagnosis. HRQoL measurements are also available 1 year after diagnosis for *n* = 214 of these patients, and 2 years after the diagnosis in *n* = 195 patients. For the patients in this sub-cohort who are still alive when this project is carried out, information will be collected about their current HRQoL using the general module of version 3.0 of the *European Organization for Research and Treatment of Cancer* (EORTC) *QLQ-C30* questionnaire [19] which is applicable to all cancer patients. We will also apply the EORTC QLQ-CR29 module which is specific to colon and rectal cancer patients [20].

### Statistical analysis

#### Overall and cancer-specific survival analysis

Overall survival will be analysed using Kaplan–Meier curves, bivariate log-rank tests, and multivariate Cox proportional-hazards models. For cancer-specific and disease-free survival, a competing risk analysis will be performed. The cumulative incidence of CRC-related death in the follow-up will be estimated considering death from other causes as a competing risk, using the method proposed by Kalbfleisch and Prentice [21]. Disease-specific mortality will be compared according to different characteristics, using Gray’s test [22]. Finally, a multivariate analysis will be performed using the model proposed by Fine and Gray [23].

#### Relative survival

Relative survival will be estimated as the ratio of observed survival of CRC patients and the survival that would have been expected if the patients had had the same mortality rate as the general population. The expected survival will be estimated from the population mortality rates estimated for the health area by age group, sex, and study year, using the Ederer II method [24]. Following other publications, the ‘statistical cure’ point will be defined as the point at which the relative survival curve plateaus, e.g., when the patients remaining alive experience the same mortality rate as the age and sex-matched population without cancer [9–13]. Thus, the value at which the plateau is achieved will provide an estimation of the curation rate.

#### Cure models

The cure rate and ‘latency’ (time to death or recurrence) will be estimated using parametric and semi-parametric mixture cure models [8]. For parametric models, logistic regression will be used to analyse the cure fraction and the Weibull distribution will be used to model the survival probability of uncured patients. For semi-parametric analysis, long-term survival will be modelled using a Cox proportional-hazards model. The following results will be obtained from the estimates of the models, (a) the cure rate; (b) relative survival curves for all the patients and for uncured patients; (c) median survival rates in uncured patients; and (d) the timepoint at which 90% of the uncured patients have died. Estimates will be obtained for each sex and CRC location (colon vs. rectum), according to age (15–49, 50–59, 60–69, 70–79, and 80–99), and year of diagnosis. Using these models, the effect of different covariates on the cure rate and survival of the uncured patients will be determined independently. The parameters of the model will be estimated using the maximum likelihood method.

#### Quality of life

The mean HRQoL questionnaire scores and the prevalence of associated symptoms will also be determined, together with their 95% confidence intervals. HRQoL will be compared with the baseline data using statistical analysis tests for paired data in order to determine the factors associated with a better HRQoL. We will test the normality of the quantitative variables using the Kolmogorov–Smirnov test and will apply Student *t*-tests or Mann–Whitney U tests as appropriate to compare numerical parameters between two groups. An analysis of variance or the Kruskall–Wallis test will be used to compare more than two groups. The association between qualitative variables will be contrasted using the chi-square statistic and associations between quantitative variables will be studied with Pearson correlation coefficients and Spearman’s rho. Linear regression and multiple logistic models will be used to control for the effect of several variables on HRQoL, perceived quality of care, and patient function. All the statistical analyses will be carried out with SPSS (version 21.0; IBM Corp., Armonk, NY) and R software for Windows.

## Discussion

CRC is a major public health problem worldwide and is one of the most common tumour types in terms of its incidence and associated mortality rates [1]. Advances in the diagnosis and treatment of CRC has caused its mortality rates to decrease in recent years, thus increasing the number of long-term CRC survivors [3]. These survivors experience the normal issues related to aging, along with the physical and emotional effects of a cancer diagnosis and of its treatments [25]. Therefore, one goal remains further improvement of the probability of ‘curing’ this disease while also improving the life expectancy for these cured patients.

Thus, large epidemiological studies are important to check the effects of diagnosis and treatment improvements in terms of patient survival, to identify prognostic factors, and to detect subgroups who could need more frequent follow-up surveillance. Therefore, the primary endpoint of this proposed study will be to provide an accurate, updated estimation of long-term survival in CRC patients, as well as to identify variables that may be associated with the probability of a cure and with survival time in uncured patients.

Traditional statistical methods (such as Kaplan–Meier curves or the Cox proportional hazards model) commonly used to estimate survival in CRC patients, implicitly assume that all patients with the same global diagnosis of CRC are at risk of developing the event of interest (e.g., death from the tumour) if they are followed for a sufficiently long period of time. This hypothesis is reasonable when overall mortality is analysed, but falls short when analysing specific-cause mortality or disease-free survival because some patients will never die from the CRC cancer or suffer a recurrence of the tumour. Therefore, alternative survival models taking this into account should be considered.

Statistical cure models could be a useful alternative in this context, even though they are not often currently used in clinical research [26]. Cure models assume that a fraction of the patients will be cured by the treatment and will never be at risk for suffering an event related to the specific disease again (e.g., CRC death, recurrence, or metastases). Therefore, these models might provide a better estimation of the ‘cure fraction’ while also modelling the average time to the occurrence of a new event among uncured patients as well as associated prognosis factors. These models therefore allow the clinical determinants of the cure and the variables associated with survival to be analysed [27].

To the best of our knowledge, this is the first study in Spain employing cure models to analyse the long-term prognosis of CRC patients. Furthermore, no other published studies have evaluated the impact of clinical variables other than age, sex, disease stage, or location on long-term CRC survival by using a similar methodology. The research published to date are mainly cancer-registry population studies that do not include clinically important variables such as those registered in hospital-based cohort studies like this one. Other strengths of this project are its relatively large sample size and the long-term follow-up periods considered.

Health care for long-term survivors must include strategies not only for the early diagnosis of recurrences and new neoplasms, but also to detect the long-term medical and psychological effects of cancer diagnosis and treatments [25]. Thus, characterisation of long-term survivors and analysis of their outcomes will help researchers to assess the adequacy of the medical care provided to them and to optimise the health resources invested in these patients. The secondary aims of this project will be to provide information on the symptoms and HRQoL of long-term CRC patients. These results will allow us to confirm whether, as other authors have indicated, HRQoL returns to normal 1 year after diagnosis [28]. This data could be important to CRC patients, for example, in planning scheduled follow-up visits to screen not only for medical issues, but also for the late effects of treatments on patient HRQoL and symptoms.

Finally, this research is not without limitations. Firstly, it is a single-centre study which includes a sample from only one hospital in Spain. Secondly, some of the measurements were obtained from clinical records, and so the possibility of information bias could not be discarded. This could limit the generalisability of the results and so, future studies including other populations are warranted. Nonetheless, this study reflects the outcomes of real-life practice in a specialised hospital. Moreover, because it is a single-centre study, the procedures, metrics, and variables collected were homogeneous. In summary, this study had a large sample size and long follow-up time and its results are expected to help identify the needs and clinical situation of long-term survivors of CRC and will be useful for proposing new models of care for the follow-up of these patients.

## Data Availability

The datasets that will be used or analysed during this current study are not yet available because the study is presently ongoing. They will be available from the corresponding author upon reasonable request once recruitment and data collection is complete.

## Abbreviations

CRC: Colorectal cancer
HRQoL: Health-related quality of life

## References

[1] Bray F, Ferlay J, Soerjomataram I, Siegel RL, Torre LA, Jemal A (2018). Global cancer statistics 2018: GLOBOCAN estimates of incidence and mortality worldwide for 36 cancers in 185 countries. CA Cancer J Clin.

[2] Siegel RL, Miller KD, Jemal A (2019). Cancer statistics, 2019. CA Cancer J Clin.

[3] De Angelis R, Sant M, Coleman MP, Francisci S, Baili P, Pierannunzio D (2014). EUROCARE-5 working group. Cancer survival in Europe 1999-2007 by country and age: results of EUROCARE--5-a population-based study. Lancet Oncol.

[4] Abdel-Rahman O (2018). Challenging a dogma: five-year survival does not equal cure in all colorectal cancer patients. Expert Rev Anticancer Ther.

[5] Hewitt M, Greenfield S, Stovall E (2006). From cancer patient to cancer survivor: lost in transition.

[6] Primrose JN, Perera R, Gray A, Rose P, Fuller A, Corkhill A (2014). Effect of 3 to 5 years of scheduled CEA and CT follow-up to detect recurrence of colorectal cancer: the FACS randomized clinical trial. JAMA..

[7] Wolbers M, Koller MT, Stel VS, Schaer B, Jager KJ, Leffondré K (2014). Competing risks analyses: objectives and approaches. Eur Heart J.

[8] Lambert PC, Thompson JR, Weston CL, Dickman PW (2007). Estimating and modeling the cure fraction in population-based cancer survival analysis. Biostatistics..

[9] Gauci D, Allemani C, Woods L (2016). Population-level cure of colorectal cancer in Malta: an analysis of patients diagnosed between 1995 and 2004. Cancer Epidemiol.

[10] Izadi N, Koohi F, Safarpour M, Naseri P, RahimiS KS (2020). Estimating the cure proportion of colorectal cancer and related factors after surgery in patients using parametric cure models. Gastroenterol Hepatol Bed Bench.

[11] Shack LG, Shah A, Lambert PC, Rachet B (2012). Cure by age and stage at diagnosis for colorectal cancer patients in north West England, 1997-2004: a population-based study. Cancer Epidemiol.

[12] Lambert PC, Dickman PW, Osterlund P, Andersson T, Sankila R, Glimelius B (2007). Temporal trends in the proportion cured for cancer of the colon and rectum: a population-based study using data from the Finnish Cancer registry. Int J Cancer.

[13] Ito Y, Nakayama T, Miyashiro I, Sugimoto T, Ioka A, Tsukuma H (2012). Trends in ‘cure’ fraction from colorectal cancer by age and tumour stage between 1975 and 2000, using population-based data, Osaka, Japan. Jpn J Clin Oncol.

[14] Hart TL, Charles ST, Gunaratne M, Baxter NN, Cotterchio M, Cohen Z (2018). Symptom severity and quality of life among long-term colorectal Cancer survivors compared with matched control subjects: a population-based study. Dis Colon Rectum.

[15] Esteva M, Ramos M, Cabeza E, Llobera J, Ruiz A, Pita S (2007). Factors influencing delay in the diagnosis of colorectal cancer: a study protocol. BMC Cancer.

[16] Pita Fernández S, Pértega Díaz S, López Calviño B, González-Santamaría P, Seoane Pillado T, Arnal Monreal F (2010). Diagnosis delay and follow-up strategies in colorectal cancer. Prognosis Implications.

[17] Pita-Fernández S, Pértega-Díaz S, López-Calviño B, Seoane Pillado T, Gago García E, Seijo Bestilleiro R (2013). Diagnostic and treatment delay, quality of life and satisfaction with care in colorectal cancer patients: a study protocol. Health Qual Life Outcomes.

[18] Schoenfeld DA (1983). Sample-size formula for the proportional-hazard regression model. Biometrics..

[19] Aaronson NK, Ahmedzai S, Bergman B, Bullinger M, Cull A, Duez NJ (1993). The European organization for research and treatment of cancer QLQ-C30: a quality-of-life instrument for use in international clinical trials in oncology. J Natl Cancer Inst.

[20] Gujral S, Conroy T, Fleissner C, Sezer O, King PM, Avery KN (2007). Assessing quality of life in patients with colorectal cancer: an update of the EORTC quality of life questionnaire. Eur J Cancer.

[21] Kalbfleisch JD, Prentice RL (1980). The statistical analysis of failure time data.

[22] Gray R (1988). A class of K-sample tests for comparing the cumulative incidence of a competing risk. Ann Stat.

[23] Fine JP, Gray R (1999). A proportional hazard model for the subdistribution of a competing risk. J Am Stat Assoc.

[24] Cleries R, Ribes J, Moreno V, Esteban L, Pareja L, Gálvez J (2006). Cálculo de la supervivencia relativa. Comparación de métodos de estimación de la supervivencia esperada. Gac San.

[25] Haggstrom DA, Cheung WY. Approach to the long-term survivor of colorectal cancer. Nekhlyudov L, ed. UpToDate. Waltham, MA: UpToDate Inc https://www.uptodate.com. Accessed 7 Sept 2020.

[26] Jia X, Sima CS, Brennan MF, Panageas KS (2013). Cure models for the analysis of time-to-event data in cancer studies. J Surg Oncol.

[27] Rahimzadeh M, Baghestani AR, Gohari MR, Pourhoseingholi MA (2014). Estimation of the cure rate in Iranian breast cancer patients. Asian Pac J Cancer Prev.

[28] Gall CA, Weller D, Esterman A (2007). Patient satisfaction and health-related quality of life after treatment for colon cancer. Dis Colon Rectum.

